# Potent Small-Molecule Inhibitors Targeting Acetylated Microtubules as Anticancer Agents Against Triple-Negative Breast Cancer

**DOI:** 10.3390/biomedicines8090338

**Published:** 2020-09-09

**Authors:** Ahreum Kwon, Gwi Bin Lee, Taein Park, Jung Hoon Lee, Panseon Ko, Eunae You, Jin Hee Ahn, Soo Hyun Eom, Sangmyung Rhee, Woo Keun Song

**Affiliations:** 1 Cell Logistics and Silver Health Research Center, School of Life Sciences, Gwangju Institute of Science and Technology, Gwangju 61005, Korea; kar3189@gist.ac.kr; 2 Department of Chemistry, Gwangju Institute of Science and Technology, Gwangju 61005, Korea; hshmhshm@gist.ac.kr (G.B.L.); taeinpark@gist.ac.kr (T.P.); jhahn@gist.ac.kr (J.H.A.); eom@gist.ac.kr (S.H.E.); 3 Department of Biochemistry and Cell Biology, Geisel School of Medicine, Dartmouth College, Hanover, NH 03755, USA; Junghoon.Lee.Gr@dartmouth.edu; 4 Department of Life Science, Chung-Ang University, Seoul 06974, Korea; kpskoh@hotmail.com (P.K.); yea108@naver.com (E.Y.)

**Keywords:** microtubule acetylation, triple-negative breast cancer, anti-cancer agent, apoptosis

## Abstract

Microtubules are one of the major targets for anticancer drugs because of their role in cell proliferation and migration. However, as anticancer drugs targeting microtubules have side effects, including the death of normal cells, it is necessary to develop anticancer agents that can target microtubules by specifically acting on cancer cells only. In this study, we identified chemicals that can act as anticancer agents by specifically binding to acetylated microtubules, which are predominant in triple-negative breast cancer (TNBC). The chemical compounds disrupted acetylated microtubule lattices by interfering with microtubule access to alpha-tubulin acetyltransferase 1 (αTAT1), a major acetyltransferase of microtubules, resulting in the increased apoptotic cell death of MDA-MB-231 cells (a TNBC cell line) compared with other cells, such as MCF-10A and MCF-7, which lack microtubule acetylation. Moreover, mouse xenograft experiments showed that treatment with the chemical compounds markedly reduced tumor growth progression. Taken together, the newly identified chemical compounds can be selective for acetylated microtubules and act as potential therapeutic agents against microtubule acetylation enrichment in TNBC.

## 1. Introduction

Breast cancer is the most frequently diagnosed cancer, and approximately 13% of women will be diagnosed with invasive breast cancer in their lifetime [1,2]. Breast cancer is a heterogeneous disease composed of several well-recognized subtypes with different clinical and prognostic characteristics [3,4]. To classify the molecular subtypes of breast cancer, the status of estrogen receptor (ER), progesterone receptor (PR), and human epidermal growth factor receptor 2 (HER2) has been considered [5], and the discrimination of these subtypes is crucial for determining treatment options and selecting anticancer agents.

Triple-negative breast cancer (TNBC), characterized by the absence of ER, PR, and HER2, represents approximately 15~20% of all breast cancers [6]; however, its metastasis and mortality are significantly worse than those of other subtypes [7]. Although various anticancer therapeutic agents that can target specific receptors in breast cancer have been developed [8], TNBC does not respond to hormonal therapies that target HER2 and ER [9]. In order to overcome the limitations of TNBC therapeutic options, platinum-based compounds, including cisplatin and carboplatin, have been used in therapeutic trials for TNBC, in which they can effectively interfere with DNA replication and related cellular processes [10,11,12,13]. In addition to the development and application of novel anticancer agents, knowledge of specific TNBC characteristics will provide more options in the selection of therapeutic trials. Therefore, it is crucial to identify therapeutic agents based on specific features for TNBC [14,15]. Microtubules are essential cytoskeletal components in eukaryotic cells, which are composed of α- and β-tubulin heterodimers arranged in the form of filamentous tubes [16], and their polymerization dynamics are firmly controlled spatially and temporally during cellular processes such as intracellular transport and cell division [17,18]. Microtubule disruption reagents such as paclitaxel and Vinca alkaloids have been successfully used as chemotherapeutic drugs against various cancers [19]; however, they can act on normal cells in other tissues, causing serious side effects such as nervous system adverse reactions [20] and abnormal changes in inflammatory cytokines in the plasma of cancer patients during chemotherapy [21,22]. Therefore, the development of anticancer agents that specifically target microtubules in cancer cells is required.

Microtubule dynamics and organization are mainly regulated through post-translational modifications (PTMs), such as detyrosination [23], glutamylation [24], and acetylation [15]. In contrast to other PTMs on the outer surface of polymerized microtubules, acetylation of the α-tubulin luminal residue lysine 40 (K40), a residue exposed to the microtubule lumen, is a well-known PTM [25,26]. In mammals, microtubule acetylation is an evolutionarily conserved modification regulated by alpha-tubulin acetyltransferase 1 (αTAT1) [27] and deacetylases including HDAC6, which is a NAD-independent deacetylase [28], and Sirt2, which is a NAD-dependent deacetylase [29]. Microtubule acetylation is considered as a hallmark of long-lived microtubules, which may be a consequence of microtubule stability rather than a contributing factor [30], and it has been reported to be involved in several biological processes related to microtubule flexibility [31], intracellular transport through kinesin-1 regulation [32], and cilia formation [33]. Furthermore, the relationship between microtubule acetylation and human diseases has revealed that the dysregulation of microtubule acetylation may be associated with Charcot-Marie-Tooth disease [34], Alzheimer’s disease [35], and heart diseases [36]. Studies have been conducted on cell mobility according to the characteristics of microtubules [37], and some studies have focused on the relationship between microtubule acetylation and cancer progression. We reported recently that acetylated microtubules are required for TGF-β-induced cancer-associated fibroblast activation in breast cancer tissues [38], suggesting that microtubule acetylation seems to be a prerequisite cellular factor for breast cancer progression and that the downregulation of microtubule acetylation is important in breast cancer therapy. In this regard, we attempted to screen small chemical inhibitors for interfering with microtubule acetylation. The chemical inhibitors identified in this study showed the potential to inhibit αTAT1, a microtubule acetyltransferase, resulting in the downregulation of microtubule acetylation. We also demonstrated that the novel inhibitors successfully downregulated tumor growth in a mouse xenograft model. Therefore, the newly identified inhibitors could function as therapeutic agents against TNBC by directly inhibiting microtubule acetylation.

## 2. Materials and Methods

### 2.1. Sample and Data Collection

Paraffin-embedded human breast cancer tissue samples and patient pathological data were obtained from US Biomax, Inc., Derwood, MD, USA (BR10011). The Gwangju Institute of Science and Technology (GIST) Institutional Review Board approved this study (20191128-BR-49-05-02, approved on 28 November 2019).

### 2.2. Cell Culture

MDA-MB-231 cells were kindly provided by Dr. Jeong-Seok Nam (GIST, Gwangju, Korea) and maintained in Dulbecco’s Modified Eagle’s Medium (high glucose; Gibco, Billings, MT, USA) containing 10% fetal bovine serum (FBS), 50 µg/mL streptomycin, and 50 units/mL penicillin. ZR-75-1, T47D, HCC-1428, HCC-1419, and Hs578T were purchased from the Korean Cell Line Bank and maintained as specified in the datasheet.

### 2.3. Generation of αTAT1 Knockout (KO) Cells via CRISPR-Cas9

αTAT1 KO using the CRISPR-Cas9 system was performed as previously described [39]. Annealed sgRNA was cloned into the plasmid lentiCRISPR v2. For lentiviral production, the cloned αTAT1-targeting vector, psPAX2, and pMD2.G were co-transfected with polyethylenimine into HEK293T cells. After 48 h of incubation, the lentivirus produced from HEK293T cells was collected in conditioned media (CM). MDA-MB-231 cells were infected with the lentivirus in the presence of polybrene (8 µg/mL) for 48 h and selected with puromycin (1 µg/mL) for 2 weeks.

### 2.4. Chemical Library Screening

A ChemBridge chemical library (NM1117-1) containing 30,000 small synthetic compounds dissolved in dimethyl sulfoxide (DMSO) as a 10 mM stock solution was used. Spin90-KO mouse embryonic fibroblasts (MEFs) with consistently high expression of acetyl-α-tubulin [40] were cultured in 96-well plates, treated with each chemical compound at 10 µM, stained with antibodies against acetyl-α-tubulin (Sigma, St. Louis, MO, USA), and finally analyzed using an IX81 microscope. Selected candidates were analyzed quantitatively by western blotting.

### 2.5. Western Blotting

Collected cells were lysed in radioimmunoprecipitation assay buffer (RIPA buffer) containing 50 mM Tris-HCl (pH 7.4), 150 mM NaCl, 1% NP-40, 1% SDS, 0.25% sodium deoxycholate, 10 mM NaF, 1 mM Na_3_VO_4_, and protease inhibitor cocktail (Roche, Switzerland). Primary antibodies against acetyl-α-tubulin (Sigma), α-tubulin (Sigma), Cyclin Antibody Sampler Kit (Cell Signaling Technology, Danvers, MA, USA), and Acetyl-Histone H3 Sampler Kit (Cell Signaling Technology, USA) were used.

### 2.6. Immunofluorescence Staining

To detect acetyl-α-tubulin expression after treatment with the compounds, MDA-MB-231 cells treated with GM-90257 and GM-90631 were fixed with 4% paraformaldehyde in PBS and permeabilized with 0.1% triton-X 100 in PBS. After blocking with 0.1% bovine serum albumin, the cells were stained with primary antibodies against acetyl-α-tubulin (Sigma). To analyze the co-localization of αTAT1 and α-tubulin, MDA-MB-231 cells transfected with Dsred2-αTAT1 were prepared in the same manner and stained with primary antibodies against α-tubulin (Sigma). To stain the microtubule bundles shown, soluble tubulin proteins were extracted by dipping into 0.1% Triton X-100 for 15 s before fixation. Cells were fixed with ice-cold methanol at −20 °C for 5 min, after which the samples were treated with blocking solution (0.1% bovine serum albumin in PBST) for 1 h. The cells were stained with primary antibodies against α-tubulin (Sigma). All fluorescence microscopy images were obtained using a confocal microscope (FV1000; Olympus, Tokyo, Japan).

### 2.7. Soft Agar Colony Formation Assay

To confirm anchorage-independent cancer cell growth, a mixture of 1% agarose solution and 2× DMEM growth media (1:1 ratio) was dispensed into 6-well plates (1.5 mL per well) and allowed to solidify at room temperature for 30 min. MDA-MB-231 cells (5 × 10^3^ cells/well) were resuspended in the growth media, mixed with 0.6% agar at 40 °C, and added to agar-coated 6-well plates. The plates were incubated at room temperature for 30 min, and 300 µL of complete growth media with chemical compounds was added to each well and incubated at 37 °C for 3 weeks.

### 2.8. Microtubule Polymerization Assay

Tubulin proteins purified from porcine brain (Cytoskeleton, Denver, CO, USA) were used for in vitro microtubule polymerization assay. The tubulin proteins were adjusted to 3 mg/mL and individually mixed with chemical compounds including paclitaxel, nocodazole, GM-90257, GM-90631, and DMSO as a control. Then, the OD value at 340 nm was determined every 30 s during the polymerization reaction at 37 °C for 1 h.

### 2.9. In Vitro Microtubule Acetylation Assay

Microtubules were purified from αTAT1 KO MDA-MB-231 cells. MTS buffer containing 80 mM PIPES, 1 mM EGTA, 1 mM MgCl_2_, 40% glycerol, 1 mM PMSF, 1 mM Na_3_VO_4_, and 10 mM NaF 10 was added to the cells for 20 min at 37 °C in an incubator. After scraping the cells in MTS buffer, paclitaxel was added to a final concentration of 20 µM. Then, the solutions were homogenized three times with a 25G syringe and incubated for 10 min at room temperature. The solutions were separated into pellets and supernatants by centrifugation at 31,100 rpm using a Ti-70 rotor (Beckman Coulter, Brea, CA, USA) for 30 min at room temperature. Pellets were re-suspended with MTS buffer containing 20 µM paclitaxel. Then, 1 mg/mL His-αTAT1, 1 µM acetyl-CoA, and microtubule acetylation inhibitors (1 mM) were added and incubated for 30 min at 37 °C. SDS sample buffer (4×) was added to stop the enzymatic activity of αTAT1, and SDS-PAGE followed by western blotting was performed.

### 2.10. Immunohistochemistry

Tumors harvested from the xenograft breast cancer mouse model were fixed in 10% formalin and embedded in paraffin blocks. Paraffin sections were deparaffinized and rehydrated with ethanol. Antigen retrieval was performed by heating in retrieval solution (IHC World, Woodstock, MD, USA) for 30 min. The following primary antibodies were used for staining: acetyl-α-tubulin (Abcam, Cambridge, UK) and Ki67 (Sigma, St. Louis, MO, USA). Nuclear counterstaining was performed with Mayer’s hematoxylin (Dako, Buffalo, NY, USA). Stained slide samples were scanned and analyzed using Aperio ImageScope (Leica Biosystems, Wetzlar, Germany).

### 2.11. Annexin V-PI Apoptosis Assay

Apoptotic and dead cells (MCF-10A, MCF-7, and MDA-MB-231) with chemical compounds were identified using an annexin V-PI apoptosis kit (BD Biosciences, San Jose, CA, USA). After 24 h of incubation with the chemical compounds, the cells were harvested and stained by FITC-annexin V and PI according to the manufacturer’s protocol. Stained cells were analyzed by flow cytometry (BD Biosciences).

### 2.12. Xenograft Tumor Inoculation

Female NOD/SCID mice (8~12 weeks) were used for the breast cancer xenograft model. MDA-MB-231 cells (1 × 10^6^ cells) mixed with Matrigel were injected into the left inguinal mammary fat pad, and developed tumors were quantified with the following equation: (V = (L × W^2^)/2, L; length, W; width). Each chemical compound was intraperitoneally injected for 15 days (once every 2 days) starting when the tumor reached 100 mm^3^. All experimental procedures with animals were performed with the approval of the Animal Care and Ethics Committees of GIST (GIST-2017-003, approved on 7 March 2018).

### 2.13. Statistical Analysis

All experimental results were the average of three repetitions, and data were analyzed by two-tailed Student’s *t*-test to confirm statistical significance between two groups (experimental and control group). The bars in graphs are shown as the mean ± standard deviation (SD), and all *p* values are two-sided. The data were regarded as statistically significant at *p* values less than 0.05: *, *p* ≤ 0.05; **, *p* ≤ 0.01; ***, *p* ≤ 0.001.

## 3. Results

### 3.1. Acetylation of α-Tubulin on K40 for TNBC Progression

**Figure 1:**
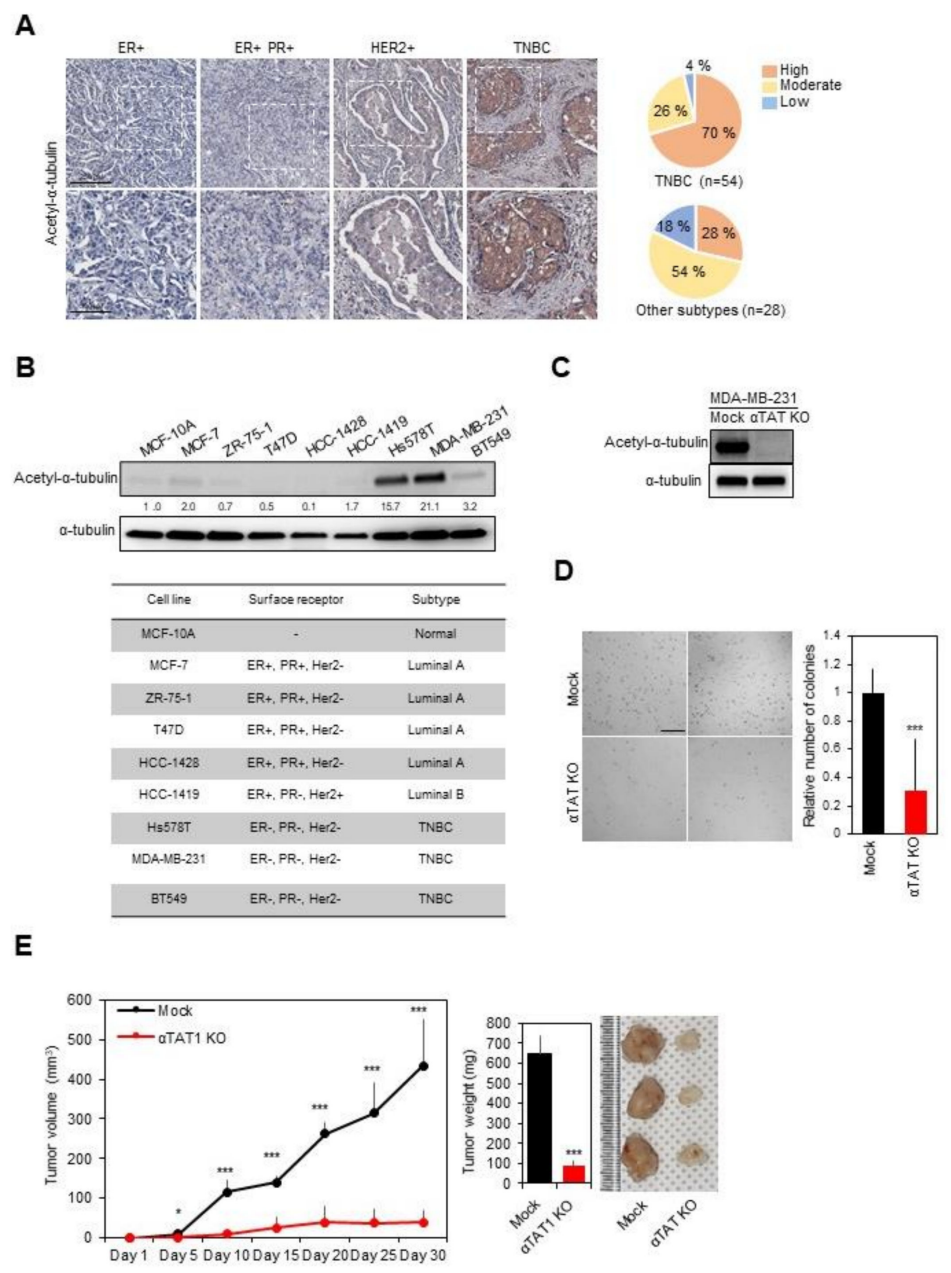
Inhibition of triple-negative breast cancer (TNBC) progression by blocking microtubule acetylation. (**A**) Representative images showing acetyl-α-tubulin expression in various subtypes of human breast cancer. Scale bar, 500 µm. The intensity was analyzed using Aperio Image Scope software. (**B**) Expression of microtubule acetylation in breast cancer cell lines (upper panel) categorized according to the subtype (lower panel). (**C**) Confirmation of acetyl-α-tubulin expression in alpha-tubulin acetyltransferase 1 (αTAT1) Knockout (KO) MDA-MB-231 cell lysates. (**D**) Anchorage-independent growth assays using αTAT1 KO MDA-MB-231 cells for 3 weeks. The number of colonies was quantified using ImageJ software. Scale bar, 1 mm. ***, *p* ≤ 0.001. (**E**) Mock and αTAT1 KO MDA-MB-231 cells were injected into the mammary fat pad of non-obese diabetic/severe combined immunodeficiency (NOD/SCID) mice (*n* = 5, each). *, *p* ≤ 0.05; ***, *p* ≤ 0.001.

To determine the level of acetyl-α-tubulin in various types of breast cancer, we obtained tissue microarray slides derived from human cancer patients and performed staining for acetyl-α-tubulin (Figure 1A). Microarray samples were separated into two groups for analysis (a group with TNBC and a group with other subtypes including ER-, PR-, and HER2-positive cases). The proportion of cases with a high expression level of acetyl-α-tubulin was significantly higher in the group with TNBC than in the group with other subtypes. We also quantitatively analyzed the expression of acetyl-α-tubulin in various breast cancer cell lines. As shown in Figure 1B, the level of acetyl-α-tubulin was significantly higher in TNBC cell lines including Hs578T, BT549, and MDA-MB-231 compared with other cell lines.

To investigate the role of acetyl-α-tubulin in TNBC, we generated a MDA-MB-231 cell line with the KO of αTAT1, a major α-tubulin acetyltransferase [23], using the CRISPR-Cas9 system (Figure 1C) and carried out anchorage-independent growth assays with mock and αTAT1 KO MDA-MB-231 cell lines. In comparison with mock cells, αTAT1 KO MDA-MB-231 cells showed a significant reduction in colony formation on soft agar (Figure 1D). Subsequently, xenograft experiments also showed that αTAT1 deficiency in MDA-MB-231 cells markedly interrupted tumor growth (Figure 1E). Overall, these results indicated that microtubule acetylation in TNBC may be related to cancer progression.

### 3.2. Screening and Optimization of Microtubule Acetylation Inhibitors

**Figure 2:**
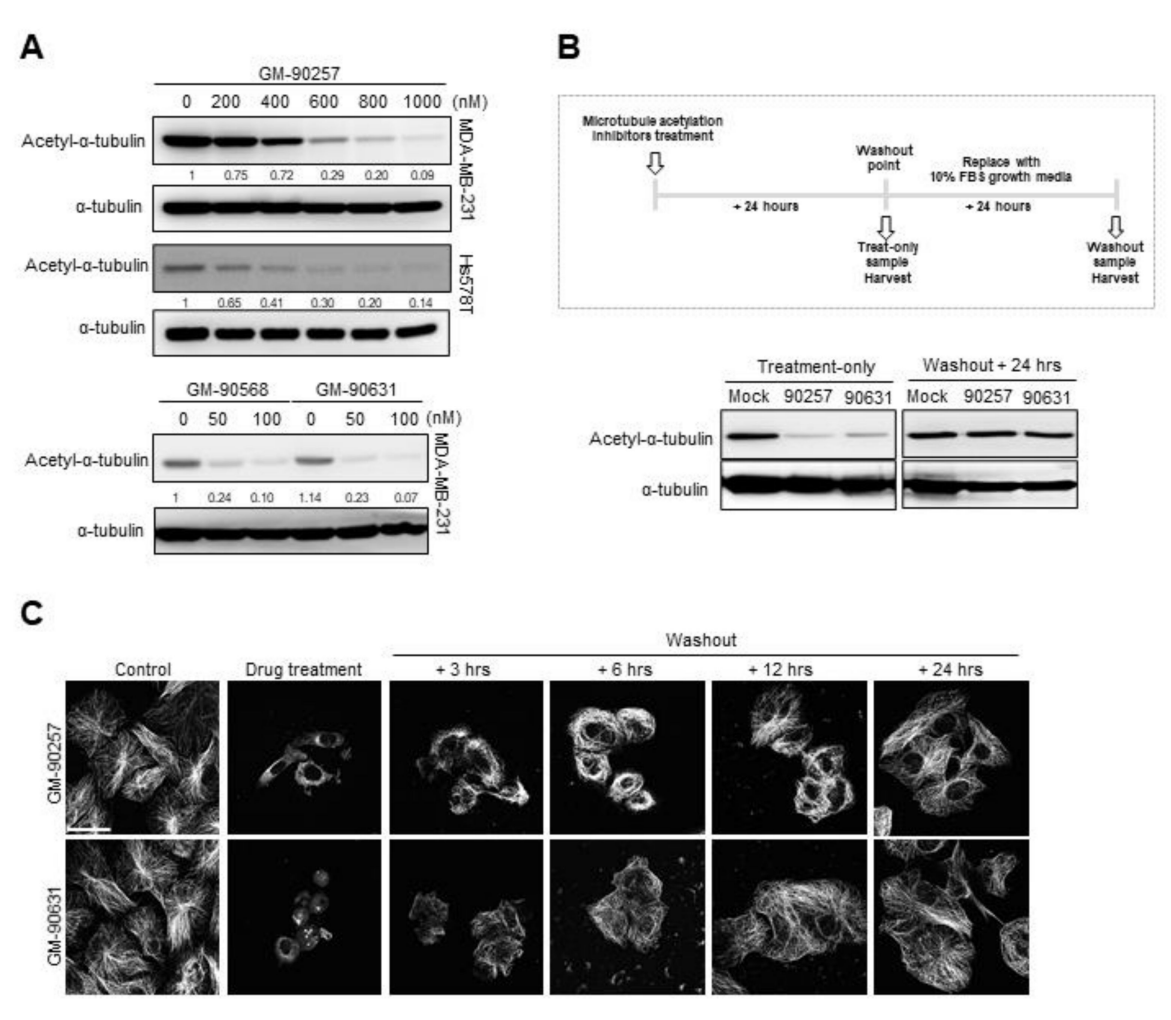
Reduction of microtubule acetylation in TNBC cell lines by selected microtubule acetylation inhibitors. (**A**) The expression of acetyl-α-tubulin in MDA-MB-231 and Hs578T cells treated with microtubule acetylation inhibitors was examined by western blotting. (**B**) GM-90257 (500 nM) and GM-90631 (50 nM) were added to MDA-MB-231 cells; one set of samples was harvested after 24 h of incubation, and the media of the other set of samples was replaced with new growth media followed by incubation for another 24 h. Western blotting for acetyl-α-tubulin was performed. (**C**) Confocal images showing the microtubules (white) in MDA-MB-231 cells treated with GM-90257 and GM-90631. Soluble tubulin in the cytoplasm was extracted before methanol fixation. After the washout of acetylation inhibitors, the cells were harvested in a time-dependent manner. Scale bar, 50 µm.

**Scheme 1:**
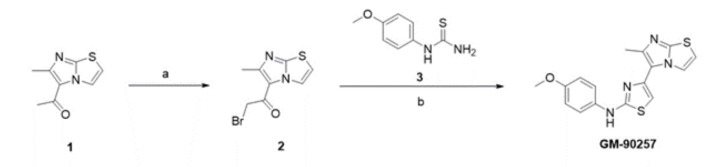
Synthesis scheme of GM-90257. Reagents and conditions: (**a**) bromine, acetic acid, reflux to room temperature, overnight; (**b**) 1-(4-methoxyphenyl)thiourea, ethanol, reflux, 20 h.

**Scheme 2:**
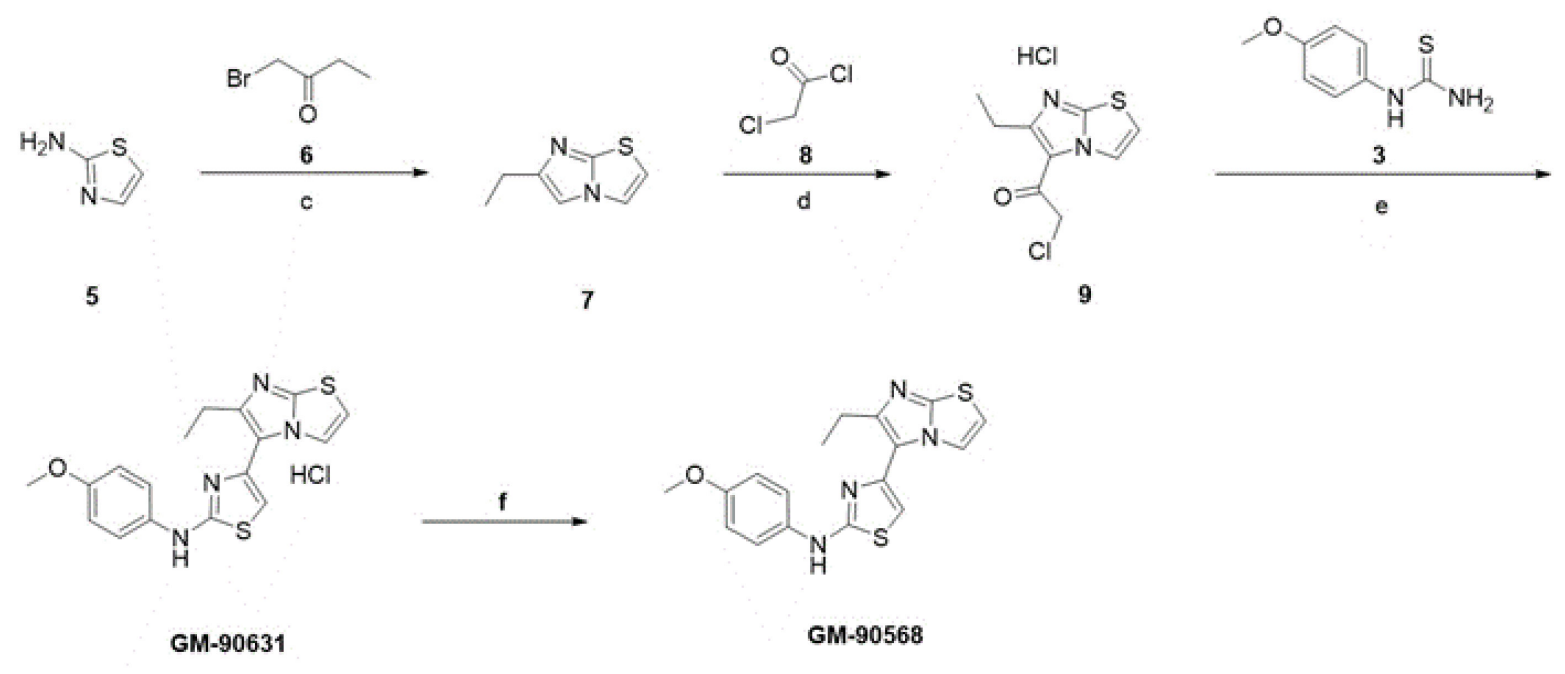
Synthesis scheme of GM-90568 and GM-90631. Reagents and conditions: (**c**) 1-bromobutan-2-one, ethanol, reflux, 18 h; (**d**) chloroacetyl chloride, 1,4-dioxane, 120 °C, 10 min; (**e**) 1-(4-methoxyphenyl)thiourea, ethanol, reflux, 20 h; (**f**) aqueous NaHCO_3_, room temperature, 30 min.

As inhibitors that directly interfere with microtubule acetylation have not been developed, we attempted to identify chemical inhibitors capable of interfering with microtubule acetylation by cell-based screening using a chemical compound library (~30,000 compounds; ChemBridge). A total of 20 potential small chemical compounds, which were selected in the first screening, were analyzed quantitatively by western blotting, and GM-90257 was identified as a hit molecule (Figure 2A). GM-90257 was optimized by the introduction of various substituents at several positions (data not shown), which identified GM-90568 and GM-90631 (HCl salt of GM-90568) as potential compounds ( Supplementary Materials Figure S1A). GM-90257 was synthesized as shown in Scheme 1. The commercially available compound **1** was converted to α-bromoketone **2**, which was coupled with thiourea **3** to give GM-90257. GM-90568 and GM-90631 were synthesized according to Scheme 2. 2-Aminothiazole **5** was reacted with α-bromoketone **6** to obtain compound **7**, followed by Friedel–Crafts acylation with chloroacetyl chloride **8** to give compound **9**. Compound **9** was cyclized with thiourea **3** to afford GM90631, which was basified by NaHCO_3_ to finally obtain GM-90568.

All three chemical compounds effectively reduced the level of acetyl-α-tubulin in MDA-MB-231 and Hs578T cells in a concentration-dependent manner. In particular, compared with GM-90257, GM-90568 and GM-90631 showed improved inhibitory effects (Figure 2A). The inhibition of acetyl-α-tubulin was abrogated within 24 h after the removal of the compounds from the cells (Figure 2B); however, there was no significant change in the level of histone H3 acetylation in the sample treated with GM-90631 (Supplementary Materials Figure S1B). Immunocytochemistry also showed that microtubule lattices were restored in a time-dependent manner after the washout of the inhibitors (Figure 2C), indicating that the inhibitors disrupted the microtubule structure. The expression of tubulin was not altered; however, both acetylated-α-tubulin and stable microtubule bundles were greatly reduced based on fluorescence microscopy (Supplementary Materials Figure S1C). Taken together, these results indicated that all three compounds could directly inhibit microtubule acetylation without any other side effects.

### 3.3. Inhibitory Effects of Potential Inhibitors on the Interaction Between α-Tubulin and αTAT1

**Figure 3:**
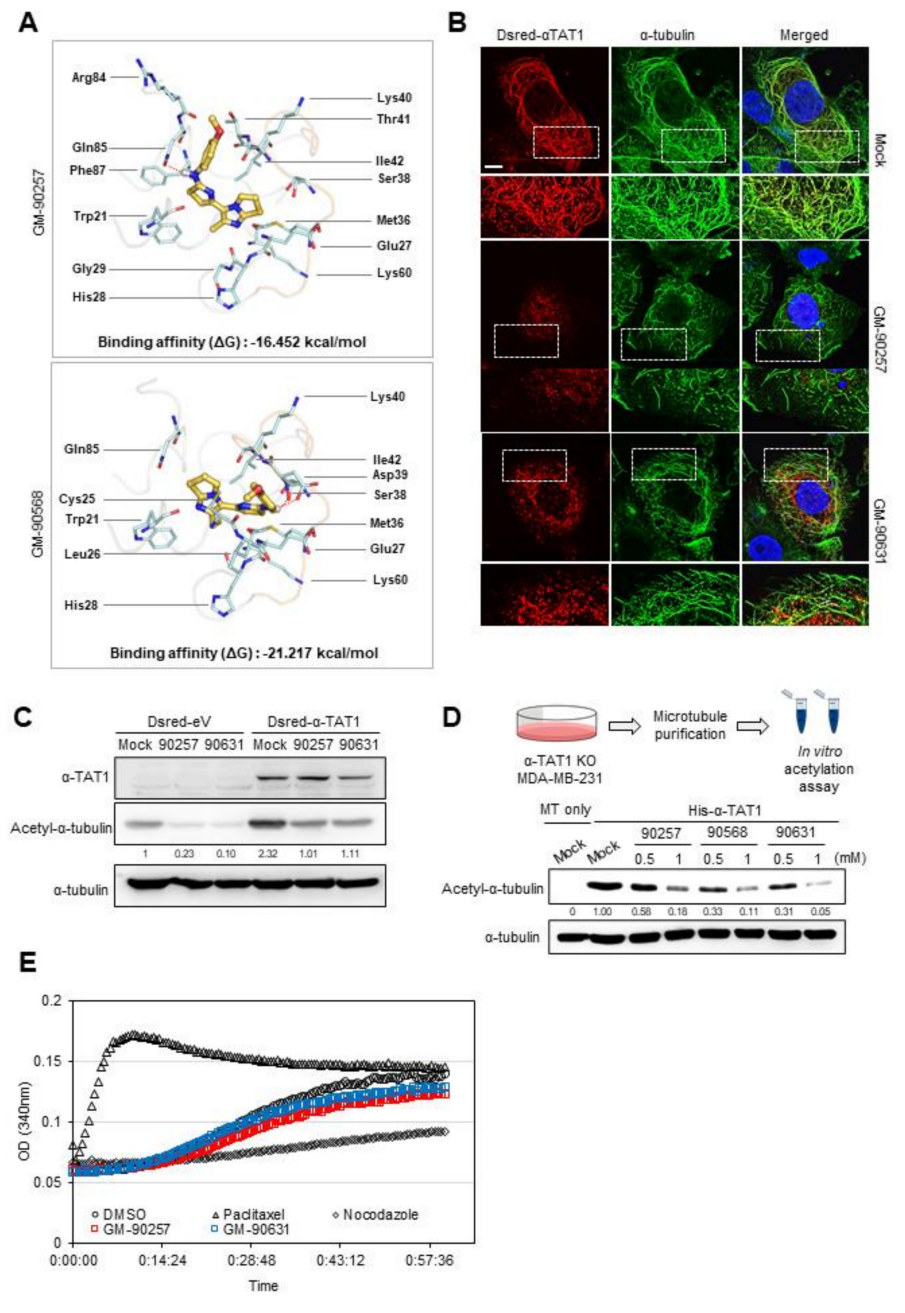
Prediction of the disruption of the interaction between microtubules and αTAT1 by microtubule acetylation inhibitors. (**A**) Predicted molecular docking models between acetylation inhibitors and the K40 residue in α-tubulin. Chemical-protein interactions and residues in the images were visualized using PyMOL program, and the binding affinity was obtained from GalaxyWEB. Hydrogen bonds are indicated by dashed red lines, and pale cyan sticks represent residues interacting with chemical compounds in α-tubulin. (**B**) Overexpressed αTAT1 (red), α-tubulin (green), and nucleus (blue) in MDA-MB-231 cells were detected by immunofluorescence staining. Scale bar, 50 µm. (**C**) DsRed-αTAT1-expressed MDA-MB-231 cell lysates were analyzed by western blotting. (**D**) Microtubules purified from αTAT1 KO MDA-MB-231 cells were incubated with His-αTAT1, acetyl-CoA, and one of the inhibitors (GM-90257, GM-90568, and GM-90631; 0.5 and 1 mM) or DMSO as a control for 30 min at 37 °C. (**E**) In vitro microtubule polymerization assay. DMSO was used as a vehicle control, and all reagents including paclitaxel, nocodazole, GM-90257, and GM-90631 were used at the same concentration of 1 µM.

In order to determine which proteins may interact with our chemical compounds to inhibit microtubule acetylation, we used GalaxyWEB to predict ligand-protein interactions. First, we performed docking simulation with the active site of HDAC6, Sirt2, and αTAT1, which are involved in microtubule acetylation, to identify the target enzyme of potential inhibitors of microtubule acetylation [23]. However, there was no significant ligand-protein docking model. Then, molecular docking simulations were performed on α-tubulin to determine whether potential acetylation inhibitors can directly bind to α-tubulin and inhibit acetylation on the K40 residue. Both the GM-90257 and GM-90568 inhibitors were docked into an identical cleft and showed reasonable affinity. According to the docking results, GM-90257 was stably bound by a hydrogen bond with Gln85 and surrounded by a hydrophobic environment with various residues. GM-905680 formed a hydrogen bond with the backbone of Ser38 and the side chain of Asp39 and was predicted to be surrounded by a hydrophobic environment in the cleft, similar to GM-90257. When both inhibitors were bound to α-tubulin, it appeared to reduce the flexibility of the loop with the acetylation site, resulting in the inhibition of acetylation (Figure 3A). In particular, the binding affinity between GM-90568 and the K40 residue was predicted to be much higher than that for GM-90257, which is consistent with the result of the previous experiment as shown in Figure 2A.

As the predicted docking model between chemical compounds and α-tubulin prevented the recruitment of αTAT1 to the K40 residue in α-tubulin, we examined the localization of DsRed-tagged αTAT1 and α-tubulin with a fluorescence microscope in the presence of GM-90257 and GM-90631. Figure 3B showed that the DsRed-αTAT1 signal was overlapped with that of the microtubule structure in the mock-treated cells. However, in the presence of the inhibitors, DsRed-αTAT1 appeared as a puncta structure around the cytosol. Interestingly, the microtubules appeared broken in the presence of the inhibitors (Figure 3B). Both inhibitors also attenuated microtubule acetylation, which was increased by αTAT1 overexpression (Figure 3C). In vitro acetylation assay using the microtubules obtained from αTAT1 KO cells confirmed that the inhibitors directly reduced microtubule acetylation (Figure 3D).

To determine whether inhibitors can affect the rate of microtubule polymerization, we performed in vitro microtubule polymerization assay using the inhibitors with other microtubule-disrupting agents. Unlike paclitaxel or nocodazole, which are known to act directly on microtubule polymerization, the polymerization rate of microtubules was not different in the presence of GM-90257 and GM-90631 compared with the control (DMSO) (Figure 3E). Taken together, these results indicated that the inhibitors directly reduced microtubule acetylation by preventing the binding of αTAT1 to the microtubules.

### 3.4. Induction of the Apoptosis of TNBC Cells by GM-90257 and GM-90631

**Figure 4:**
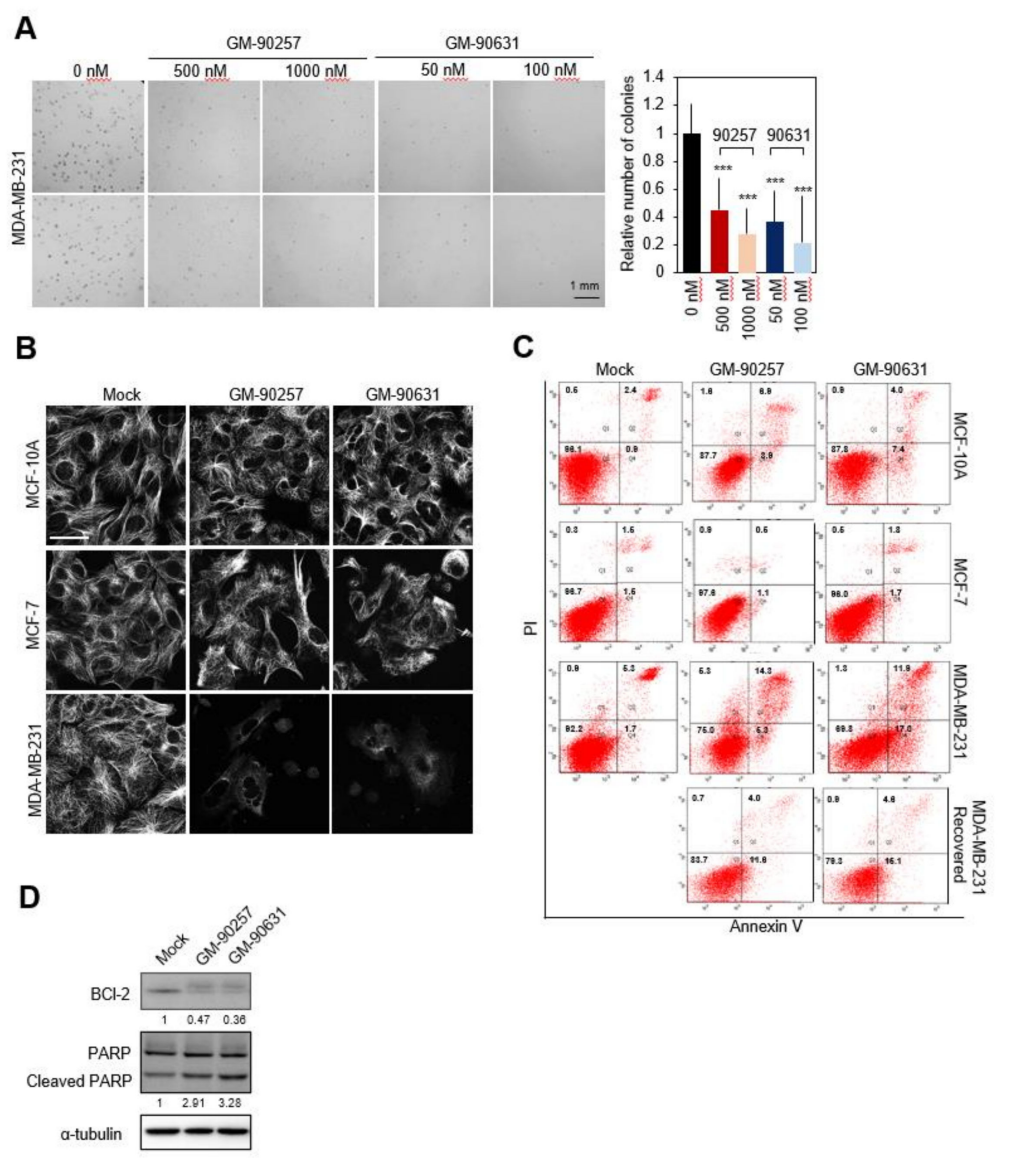
Induction of the apoptosis of TNBC cells by microtubule acetylation inhibitors. (**A**) Anchorage-independent growth assays using MDA-MB-231 cells treated with GM-90257 (500 and 1000 nM) and GM-90631 (50 and 100 nM) for 3 weeks. The number of colonies was quantified using ImageJ software. Scale bar, 1 mm. *** *p* ≤ 0.001. (**B**) Confocal images showing microtubules (white) in MCF-10A, MCF-7, and MDA-MB-231 cells after GM-90257 (500 nM) and GM-90631 (50 nM) treatment. Scale bar, 50 µm. (**C**) MCF-10A, MCF-7, and MDA-MB-231 cells treated with GM-90257 (500 nM) and GM-90631 (50 nM) and drug-eliminated MDA-MB-231 cells were double-stained with annexin V and propidium iodide (PI) and analyzed by flow cytometry. The four populations of cells in the quadrants moving clockwise are as follows: non-apoptotic dead cells (Q1), late apoptotic cells (Q2), viable cells (Q3), and early apoptotic cells (Q4). (**D**) The expression levels of BCl-2, PARP, and cleaved PARP in MDA-MB-231 cells treated with GM-90257 (500 nM) and GM-90631 (50 nM) were examined by western blotting.

To clarify the functional effects of GM-90257 and GM-90631 in MDA-MB-231 cells, we performed anchorage-independent growth assays for 2 weeks with the chemical compounds. Both compounds significantly reduced the colony formation of MDA-MB-231 cells in a concentration-dependent manner (Figure 4A).

The previous result showed that the chemical compounds inhibited microtubule acetylation concomitant with the disruption of microtubule lattices (Figure 2C). Therefore, we examined whether the inhibitors can disrupt microtubule lattices by acting specifically on acetylated microtubules. Immunocytochemistry revealed that the microtubule structure in MDA-MB-231 cells was specifically disrupted by the inhibitors (Figure 4B). The result demonstrated that the inhibitors could act exclusively on acetylated microtubules considering the higher level of acetylated microtubules in untreated MDA-MB-231 cells compared with untreated MCF-10A and MCF-7 cells.

As the inhibitors specifically disrupted the microtubule structure of MDA-MB-231 cells, we investigated whether cell death caused by the inhibitors is also limited to MDA-MB-231 cells through FACS analysis. As shown in Figure 4C, treatment of the MCF-10A and MCF-7 cells having a low level of microtubule acetylation did not induce cell death; however, MDA-MB-231 cells showed increased apoptotic cell death following inhibitor treatment. Apoptotic cell death induced by the inhibitors was significantly reduced when the inhibitors were removed from MDA-MB-231 cells. In addition, GM-90257 and GM-90631 activated poly ADP ribose polymerase (PARP) cleavage in MDA-MB-231 cells but downregulated the anti-apoptotic protein, B-cell lymphoma (BCl-2) (Figure 4D). Taken together, these results demonstrated that the inhibitors could act specifically on cells with a high level of microtubule acetylation, leading to cell death through the disruption of microtubules.

### 3.5. Inhibition of TNBC Progression In Vivo by Blocking Microtubule Acetylation

**Figure 5:**
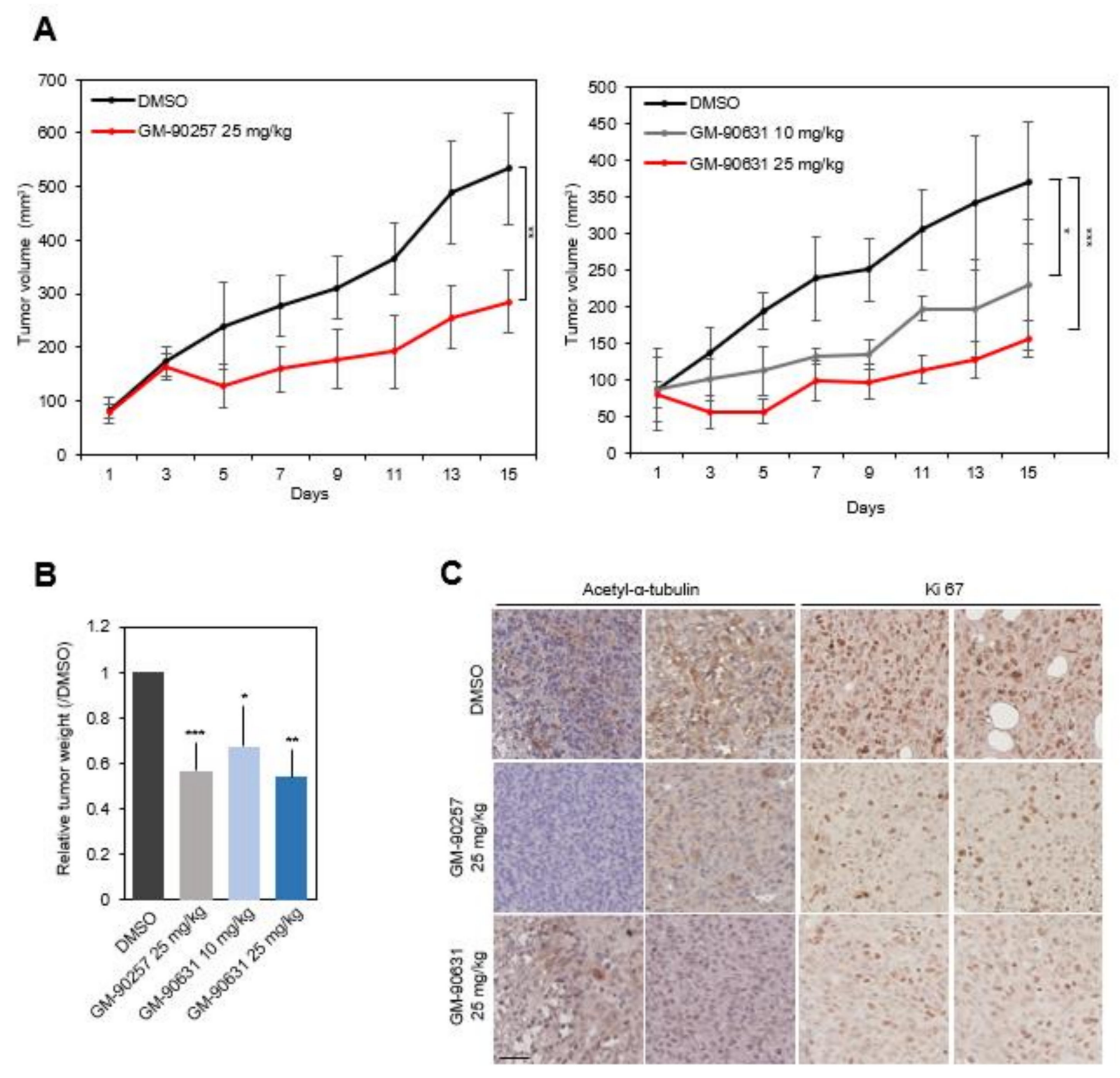
Inhibition of TNBC progression in vivo by GM-90257 and GM-90631. (**A**) MDA-MB-231 cells were injected into the mammary fat pads of NOD/SCID mice. The mice were randomly divided into two groups: control (DMSO) and 25 mg/kg GM-90257 (*n* = 5 per group, left graph). Each drug was intraperitoneally (i.p.) administered once every 2 days for 15 days. The right graph shows the experimental results for GM-90631, and the mice were randomly divided into three groups: control (DMSO), 10 mg/kg GM-90631, and 25 mg/kg GM-90631 (*n* = 5 per group). The graphs show the tumor volume growth for 15 days, and the bars in the graphs indicate the standard deviation. ** *p* ≤ 0.01. (**B**) Harvested tumor weight. * *p* ≤ 0.05; *** *p* ≤ 0.001. (**C**) Immunohistochemical analysis of acetyl-α-tubulin and Ki67 in sectioned, paraffin-embedded tumor tissues. Scale bar, 100 µm.

To evaluate the anticancer effects of the inhibitors in vivo, we injected the inhibitors into tumors in the mammary fat pad of NOD/SCID mice, which were generated from MDA-MB-231 cells. Preliminary experiments to determine the effective concentration of GM-90257 and GM-90631 in vivo indicated biocompatibility in the range of 10~50 mg/kg. GM-90257 had no anticancer effects at 10 mg/kg in vivo, and mice administered with a dose of 50 mg/kg had a mortality rate of almost 50% when the drug injection was maintained for 2 weeks (data not shown). Based on these results, we decided to inject GM-90257 at 25 mg/kg. GM-90631, which was effective at a much lower concentration in vitro, inhibited tumor formation even at 10 mg/kg. After the tumor volume reached 100 mm^3^ (average of 2 weeks after the injection of cancer cells), the mice were intraperitoneally (i.p.) administered GM-90257 and GM-90631 for 15 days; control mice were injected with DMSO once every 2 days (Supplementary Materials Figure S2A). The growth of tumors was significantly reduced in mice injected with GM-90257 and GM-90631 compared with mice injected with DMSO (Figure 5A), and there was no difference in the average weight of mice during the treatment period (Supplementary Materials Figure S2B). After treatment completion, tumors were separated from the mice, and the final tumor volume and weight were markedly reduced in a concentration-dependent manner (Figure 5B and Supplementary Materials Figure S2C). In addition, immunohistochemistry showed that the expression levels of acetyl-α-tubulin and Ki67, a representative proliferation marker, in sectioned tumor tissues were markedly decreased after treatment with GM-90257 and GM-90631 (Figure 5C).

## 4. Discussion

Although TNBC usually affects younger patients, the overall five-year survival rate for TNBC is around 77% versus 93% for other breast cancer types, and almost all women with metastatic TNBC will ultimately die from their disease [41]. For these reasons, the development of effective targeted therapeutic agents against TNBC has been actively conducted. In addition, studies have focused on specific features found in TNBC considering that anticancer agent efficacy is often diverse due to the molecular heterogeneity of TNBC with restricted treatment options [42]. For example, PARP inhibitors and platinum salt drugs are one of the most powerful anticancer agents but show different effects against TNBC depending on BRCA gene activation [43]. In order to address this problem, combined treatments with BET inhibitors regulating BRCA genes have been proposed [43]. Some studies of combined treatments using anticancer agents that can complement each other have been conducted. Here, we attempted to investigate significant factors that could be novel molecular features of TNBC and obtain a proof-of-principle for the development of novel agents against TNBC. In the study, we demonstrated that microtubule acetylation was specifically increased in TNBC patient tissues and a TNBC cell line (MDA-MB-231), and the inhibition of microtubule acetylation in MDA-MB-231 cells by newly developed chemical inhibitors markedly reduced cancer progression in vitro and in vivo.

Microtubule acetylation is known to act as a double-edged sword in cancer research and treatment. The high expression HDAC6 has been suggested to promote the proliferation of lung adenocarcinoma cells [44], and HDAC6 inhibitors have been considered as effective anticancer agents against non-small cell lung cancer [45]. Moreover, another HDCA6 inhibitor, ACY-1215, could enhance anticancer activity against colorectal cancer [46]. On the other hand, the expression of microtubule acetylation is closely associated with several cancers. Acetyl-α-tubulin is considered as a prognostic marker of squamous cell carcinoma of the head and neck especially in metastatic cases [47]. As mentioned previously, microtubule acetylation is enhanced in metastatic breast cancer (especially in basal-like breast cancer types), promotes the formation of microtentacles (which are microtubule-based membrane protrusions), and contributes to cell migration [48]. As basal-like breast cancer frequently overlaps with the TNBC subtype, it provides strong supportive evidence for TNBC.

Although GM-90257, GM-90568, and GM-90631 are not direct inhibitors of the αTAT1 active site, they can be considered as competitive inhibitors as they inhibit αTAT1 from binding to the microtubules (Figure 3). Chemical compounds with a molecular weight of less than 900 Da are defined as small molecules, and over 100 small chemical compounds have been approved as oncology drugs by the FDA to date [49]. All of the three compounds identified in this study have a molecular weight of less than 900 Da; thus, they can be classified as small compounds (GM-90257: 342.4 Da; GM-90568: 356.4 Da; GM-90631: 392.9 Da). The biggest advantage of small compounds is that they can rapidly diffuse across cell membranes and localize in intracellular spaces easily. When TNBC cells were directly treated with our compounds, both microtubule acetylation and cancer cell proliferation were effectively inhibited. In addition, it was confirmed that the chemical backbone structure of GM-90568 and GM-90631 has a much higher binding affinity with microtubules (GM-90257: -16.452; GM-90568, GM-90631: -21.217); thus, they function effectively at lower concentrations. Furthermore, the results of in vivo experiments were statistically significant. Most small-molecule anticancer drugs currently in clinical use lack tumor selectivity and have various toxic side effects [50]. Based on the in vitro and in vivo results, the selected chemical compounds demonstrated potential as anticancer agents against TNBC.

Microtubules are cytoskeletal proteins, which play an essential role in cell survival, and they have attracted interest as a target in early anticancer drug development [22]. Microtubules consist of the α, β, γ, δ, and ε families of tubulins; tubulins γ, δ, and ε are typically located in the centrosome [51], and α- and β-tubulins are the main building blocks of microtubules. Established microtubule-targeting agents (MTAs) can be divided into two groups: stabilizers and destabilizers [19]. Paclitaxel and nocodazole, which were used in the in vitro microtubule polymerization assay (Figure 3E), are representative MTAs. Paclitaxel binds to the second globular domain of β-tubulins and stabilizes tubulin dimers without GTP binding, and nocodazole induces microtubule destabilization and disrupts mitotic spindles by binding to β-tubulins [52]. Similar to paclitaxel and nocodazole, acetylation inhibitors are thought to function by directly binding to microtubules. The microtubule bundles in MDA-MB-231 cells were reduced after treatment with inhibitors of α-tubulin acetylation, similar to the effect of destabilizing agents (Figure 4B). As microtubule acetylation plays a key role in maintaining long-lived microtubules [15], microtubule bundles would not be maintained when acetylation is inhibited in cells with constitutive acetyl-α-tubulin expression, such as MDA-MB-231 cells. The findings indicated that normal cells, or other carcinoma cells, which do not express acetyl-α-tubulin constitutively, would show a weaker response compared with that of acetyl-α-tubulin-expressing TNBC cells. Although it is not known why the microtubules in MCF-10A or MCF-7 cells were resistant to acetylation inhibitor treatment, other PTMs may participate in microtubule bundle formation in MCF-10A and MCF-7 cells. Therefore, the tested acetylation inhibitors might not significantly cause microtubule bundle disruption in MCF-10A and MCF-7 cells. Furthermore, there were significantly less apoptotic MCF-10A and MCF-7 cells than apoptotic MDA-MB-231 cells when the cells were treated with microtubule acetylation inhibitors at the same concentration (Figure 4C).

Thus far, both microtubule stabilizers and destabilizers are known to act as anticancer agents by regulating cell cycle events, such as mitosis. At a high concentration, stabilizers inhibit the depolymerization or destabilization of microtubules, thus, inducing gap 2 phase (G2)/mitosis(M) arrest, which would result in growth inhibition and cell death [53]. On the other hand, a high concentration of Vinca alkaloids (powerful destabilizers) promotes microtubule depolymerization, leaving the cancer cells blocked in mitosis with condensed chromosomes due to mitotic spindle degradation [54]. In our study, a high concentration of microtubule acetylation inhibitors was used (1 µM for GM-90257 and 100 nM for GM-90631), and after 12 h, it was observed that the cells were rounded for around 48 h (data not shown). However, in the case of αTAT1 KO MDA-MB-231 cells, where microtubule acetylation is not constitutive, the duration of proliferation was longer compared with that of mock MDA-MB-231 cells in a soft environment or in vivo (Figure 1). In addition, there was no critical defect in cell proliferation when normal breast cells (MCF-10A) and luminal-subtype breast cancer cells (MCF-7) were treated with microtubule acetylation inhibitors. Based on our experimental results, it is likely that the induction of apoptosis by microtubule acetylation inhibitors does not occur by merely blocking the cell cycle, and there might be unknown molecular mechanisms that contribute to apoptosis through the regulation of microtubule acetylation. In the case of MTAs, a different effect was observed at a low concentration compared with a high concentration; for example, when paclitaxel was added at a low concentration, it arrested cells in the G1 phase but not the M phase and inhibited cancer proliferation by increasing p53 and p21 expression levels [55]. A notable result of this study is that BCl-2 was significantly inhibited after treatment with microtubule acetylation inhibitors (Figure 4D). BCl-2 is one of the potent anti-apoptotic proteins in cells. BCl-2 family members are often amplified during carcinogenesis, and it may also cause MTA resistance; thus, BCl-2 regulation may be necessary to improve sensitivity to MTAs [56]. BCl-2 expression regulation by microtubule acetylation inhibitors should be explored in subsequent studies; nevertheless, it is expected that there would be no resistance problem with the effective inhibition of BCl-2 expression. Moreover, microtubule disruption is known to effectively reduce anticancer drug resistance caused by the long-term administration of Taxol [57]. In this study, treatment with microtubule acetylation inhibitors showed that microtubule bundles were disrupted in acetylation-enriched cells; thus, they could be used in combination therapy under the appropriate conditions.

## 5. Conclusions

It was confirmed that microtubule acetylation was specifically increased in TNBC compared with other subtypes of breast cancer through the analysis of tissue microarray slides derived from breast cancer patients and human breast cancer cell lines. In addition, the disruption of acetyl-α-tubulin by αTAT1 KO significantly reduced cancer progression in vitro and in vivo. We identified three chemical compounds (GM-90257, GM-90568, and GM-90631) that could effectively inhibit microtubule acetylation. Through molecular docking simulation analysis, all three small chemical compounds were found to bind directly to α-tubulin and prevent the recruitment of αTAT1 to the K40 residue in α-tubulin. In addition, treatment with these chemical inhibitors attenuated tumor growth in vivo and caused the apoptosis of MDA-MB-231 cells (TNBC cells) with weaker effects on MCF-10A or MCF-7 cells, which have a relatively low level of microtubule acetylation. Overall, we demonstrated the potential of the microtubule acetylation inhibitors GM-90257, GM-90568, and GM-90631 as targeted therapeutic agents against microtubule acetylation enrichment in TNBC.

## Supplementary Materials

The following are available online at https://www.mdpi.com/2227-9059/8/9/338/s1, Figure S1: (A) Schematic flowchart for small chemical compound screening. (B) Acetyl-histone-H3 and total H3 expression in MDA-MB-231 cell lysates treated with GM-90631. (C) Fluorescence microscopy images showing microtubule bundles or β-tubulin and acetyl-α-tubulin in MDA-MB-231 cells after treatment with GM-90257 (500 nM) and GM-90631 (50 nM); Figure S2: (A) Photographs of mice after treatment with 25 mg/kg GM-90257 for 15 days. (B) The weight of mice showed no significant changes during treatment with GM-90257 (top) and GM-90631 (bottom). (C) Photographs of harvested tumors at the end of drug administration.
