# Supplementary material for: Potent Small-Molecule Inhibitors Targeting Acetylated Microtubules as Anticancer Agents Against Triple-Negative Breast Cancer
Source: Biomedicines. 2020 Sep 9;8(9):338. doi: 10.3390/biomedicines8090338 (PMC7555225; doi:10.3390/biomedicines8090338)
Supplement: Supplementary file 1 [file biomedicines-08-00338-s001.pdf]

biomedicines

Potent Small-Molecule Inhibitors Targeting Acetylated Microtubules as Anticancer Agents Against Triple-Negative Breast Cancer

Ahreum Kwon [1,†], Gwi Bin Lee [2], Taein Park [2], Jung Hoon Lee [3], Panseon Ko [4], Eunae You [4], Jin Hee Ahn [2], Soo Hyun Eom [2], Sangmyung Rhee [4,*] and Woo Keun Song [1,*]

[1] Cell Logistics and Silver Health Research Center, School of Life Sciences, Gwangju Institute of Science and Technology, Gwangju 61005, Korea; kar3189@gist.ac.kr

[2] Department of Chemistry, Gwangju Institute of Science and Technology, Gwangju 61005, Korea; hshmhshm@gist.ac.kr (G.B.L.); taeinpark@gist.ac.kr (T.P.); jhahn@gist.ac.kr (J.H.A.); eom@gist.ac.kr (S.H.E.)

[3] Department of Biochemistry and Cell Biology, Geisel School of Medicine, Dartmouth College, Hanover, NH 03755, USA; Junghoon.Lee.Gr@dartmouth.edu

[4] Department of Life Science, Chung-Ang University, Seoul 06974, Korea; kpskoh@hotmail.com (P.K.); yea108@naver.com (E.Y.)

* Correspondence: Sangmyung.rhee@cau.ac.kr (S.R.); wksong@gist.ac.kr (W.K.S.); Tel.: +82-62-715-2560 (S.R.); Tel.: +82-2-820-5818 (W.K.S.)

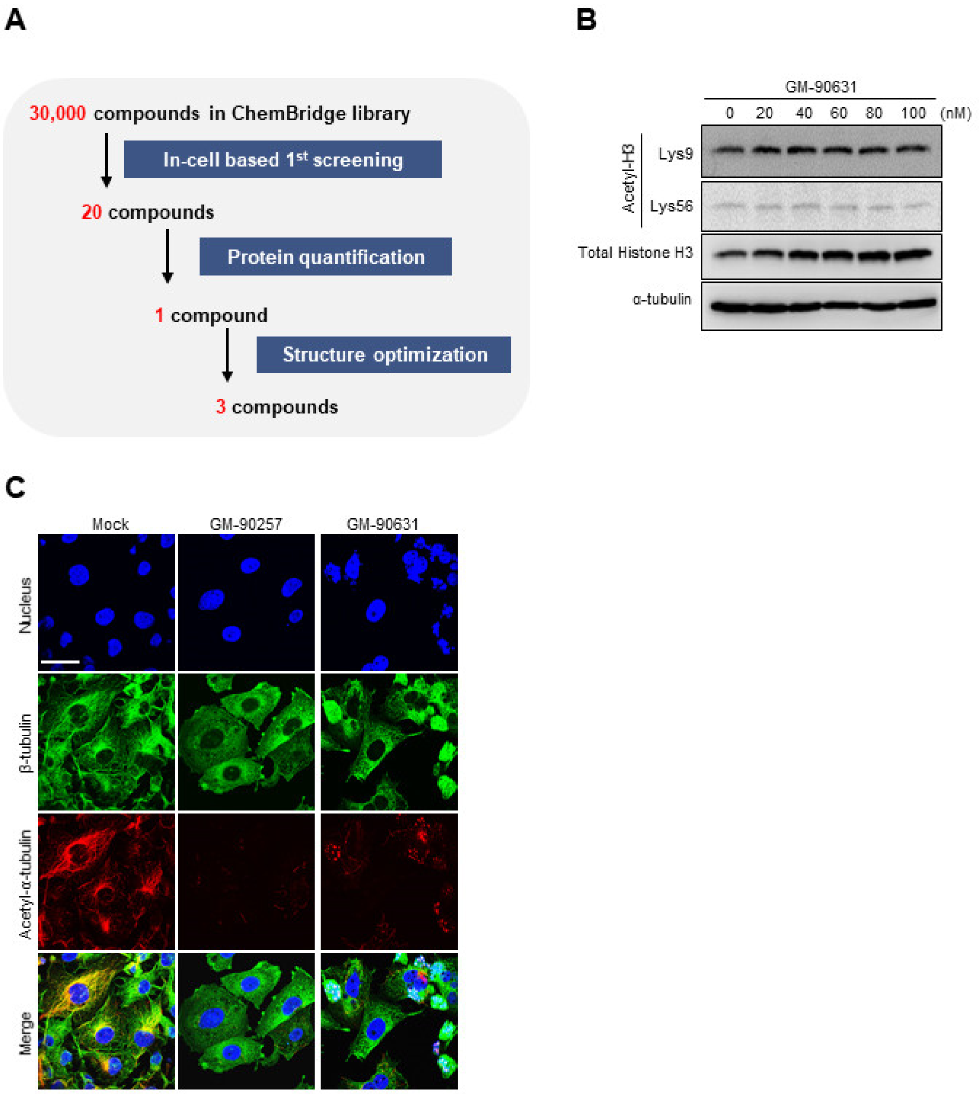

Supplementary Figure S1 (**A**) Schematic flowchart for small chemical compound screening. (**B**) Acetyl-histone-H3 and total H3 expression in MDA-MB-231 cell lysates treated with GM-90631. (**C**) Fluorescence microscopy images showing microtubule bundles or β-tubulin and acetyl-α-tubulin in MDA-MB-231 cells after treatment with GM-90257 (500 nM) and GM-90631 (50 nM).

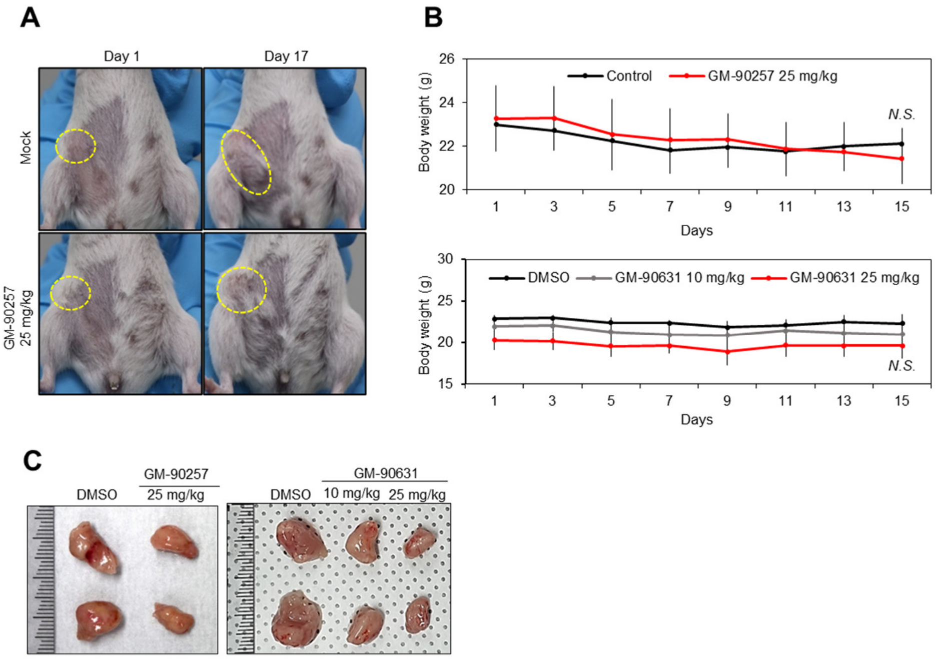

Supplementary Figure S2 (**A**) Photographs of mice after treatment with 25 mg/kg GM-90257 for 15 days. (**B**) The weight of mice showed no significant changes during treatment with GM-90257 (top) and GM-90631 (bottom). (**C**) Photographs of harvested tumors at the end of drug administration.
